# Implementación de la Política de Atención Integral en Salud en Cundinamarca, Colombia 2021

**DOI:** 10.15446/rsap.V25n6.111389

**Published:** 2023-11-01

**Authors:** Fabio Alberto Escobar-Díaz

**Affiliations:** 1 FE: Soc. M. Sc.; Ph. D. Salud Pública. Fundación Universitaria del Área Andina. Bogotá, Colombia. fescobarl3@areandina.edu.co Fundación Universitaria del Área Andina Salud Pública Fundación Universitaria del Área Andina Bogotá Colombia fescobarl3@areandina.edu.co

**Keywords:** Política de salud, atención integral de salud, integralidad en salud, Colombia *(fuente: DeCS, BIREME)*, Health policy, comprehensive health care, integrality in health, Colombia *(source: MeSH, NLM)*

## Abstract

**Objetivo:**

La Política de Atención Integral en Salud (PAIS) tiene como propósito mejorar las condiciones de salud de la población colombiana para garantizar el derecho a la salud. El objetivo de estudio fue caracterizar la implementación de esta política en el departamento de Cundinamarca en el año 2021.

**Métodos:**

Diseño cualitativo y transversal basado en entrevistas individuales semiestructuradas a representantes de entidades del sector salud a nivel nacional, departamental y municipal. La información fue codificada y categorizada mediante el análisis temático.

**Resultados:**

La PAIS y su modelo operacional fueron reajustados con la llegada del nuevo gobierno nacional en el 2018. Además, en el 2020 con los nuevos gobernantes locales, la PAIS tuvo que reposicionarse en sus agendas políticas. Debido a la pandemia, muchas actividades fueron suspendidas, entre ellas la PAIS, debido a las restricciones epidemiológicas. El modelo operacional de la PAIS carece de un lineamiento que oriente su implementación; solo se cuenta con algunas normas específicas para las rutas integrales de atención en salud y las redes integradas de servicios de salud, lo que facilitó algunos avances. Existe una escasa articulación entre la PAIS y otros Instrumentos de planeación nacional y local.

**Conclusiones:**

La implementación de la PAIS en Cundinamarca cuenta con un proceso aún incipiente, caracterizado por circunstancias a nivel político (cambios de gobierno), epidemiológico (Covid-19) e incluso operativo (falta de lineamientos específicos). El avance en la ejecución de la política podría mejorar al superarse algunas de estas circunstancias.

## Abstract (en)

**Objective:**

The Comprehensive Health Care Policy (CHCP) aims to improve the health conditions of the Colombian population to guarantee the right to health. The study's objective was to characterize the implementation of CHCP in the department of Cundinamarca in 2021.

**Methods:**

Qualitative and cross-sectional design based on semi-structured individual ¡nterviews with representatives of health sector entities at the national, departmental, and municipal levels. The information was coded and categorized through thematic analysis.

**Results:**

CHCP and its operational model were readjusted with the arrival of the new national government in 2018. Additionally, in 2020, with the new local leaders, CHCP had to reposition itself in their political agendas. Due to the pandemic, many activities were suspended, including CHCP, because of epidemiological restrictions. The operational model of CHCP lacks guidance to direct its implementation; there are only some specific norms for comprehensive health care routes and integrated health service networks, which facilitated some progress. There is a poor coordination between CHCP and other national and local planning instruments.

**Conclusions:**

The implementation of CHCP in Cundinamarca has a still incipient process, characterized by circumstances at the political (changes in government), epidemiological (Covid-19), and even operational levels (lack of specific guidelines). Progress in policy execution could improve by overcoming some of these circumstances.

El Sistema General de Seguridad Social en Salud (SGSSS) de Colombia 1 tiene como uno de sus logros más visibles el aumento en la cobertura de afiliación superior al 95 %; sin embargo, este resultado no ha estado acompañado por el acceso efectivo y oportuno a los servicios de salud, debido a diferentes barreras que no han sido superadas con las reformas que ha tenido el sistema en el 2007 y en el 2011 2-4.

Diferentes estudios realizados en Colombia 2,5-7 han abordado la problemática del acceso a los servicios de salud en el SGSSS. Ante esta situación, la Ley Estatutaria 1751 del 2015 posicionó a la salud como un derecho fundamental que debe garantizarse y, en consecuencia, estableció que el Estado debía contar con una política en salud de carácter integral que respete, proteja y garantice este derecho, incluido el acceso a los servicios de salud de manera oportuna, eficaz y con calidad 8.

De esta ley estatutaria nació la Política de Atención Integral en Salud (PAIS), adoptada en el 2016 9,10. Su objetivo ha sido garantizar el derecho a la salud de las personas en Colombia, mediante el fortalecimiento de la regulación del sistema de salud y con base en unos acuerdos que integren los objetivos del sistema de salud con los de la seguridad social 10.

La PAIS cuenta con un marco estratégico conformado por la Atención Primaria en Salud (APS), el cuidado de la salud, la Gestión Integral del Riesgo en Salud (GIRS) y el enfoque diferencial territorial y poblacional (11). Adicionalmente, cuenta con un modelo operacional denominado Modelo de Acción Integral Territorial (MAITE), aprobado en el 2019 y que pretende mejorar su implementación en las entidades territoriales (ET) y fortalecer la articulación entre los actores del SGSSS 12.

Las ET, como lo son los departamentos, los municipios y los distritos, en el marco de la descentralización en salud, tienen una gran responsabilidad en la implementación de la PAIS y su modelo operacional. El MAITE establece que los territorios ejercen el liderazgo en este proceso de implementación y seguimiento de la política, por lo cual deben realizar acciones específicas para su desarrollo mediante la coordinación con otros actores y la incorporación de la política en los planes territoriales de salud (PTS), instrumentos esenciales de planeación del sector en las ET 12.

Si se tienen en cuenta las disposiciones normativas para la implementación de la PAIS, es fundamental generar nuevos conocimientos sobre este proceso en las ET. Este estudio tuvo como objetivo comprender el estado de la implementación de esta política en el departamento de Cundinamarca, Colombia, durante el año 2021.

## MÉTODOS

Diseño cualitativo, descriptivo y transversal, por medio de un estudio de caso como método de investigación que aborda un determinado fenómeno a profundidad en su contexto real 13. El caso fue el estado de la implementación de la PAIS en el departamento de Cundinamarca, comprendiendo este fenómeno desde el nivel nacional, departamental y municipal. Por consiguiente, la muestra fue intencional o propositiva, con el fin de encontrar información a profundidad. Participaron en el estudio actores del nivel nacional, como el Ministerio de Salud y Protección Social (MSPS), y departamental, como la Secretaría de Salud de Cundinamarca (SSC). Además, se eligieron tres municipios ubicados en la ET departamental que tienen tamaños poblacionales y capacidades económicas e institucionales diferentes: Facatativá, Sibaté y Ubaté (Tabla 1).

**Tabla 1:** Actores participantes

Actor nacional	Actor departamental	Actor municipal
Ministerio de Salud y Protección Social( MSPSA - MSPSB)	Secretaría de Salud de Cundinamarca (SSC)	Ubaté
		Facatativá
		Sibaté

Se aplicaron seis entrevistas individuales semiestructuradas a los funcionarios con responsabilidades relacionadas con la PAIS en los tres niveles (nacional, departamental y municipal). Se aplicaron consentimientos informados donde se contó con su autorización para la grabación de los encuentros, que se realizaron de forma virtual mediante la plataforma Google Meet. El estudio se ajusta a los principios éticos de la Declaración de Helsinki y de las normas científicas de la investigación en salud del MSPS, al garantizarse la confidencialidad y la intimidad de la información personal de los participantes; así mismo, fue una investigación clasificada como sin riesgo 14. Las grabaciones de las entrevistas fueron transcritas en su totalidad para su análisis, mediante la generación de códigos y categorías o temas (Tabla 2). 

**Tabla 2:** Temas y sus definiciones

Tema	Definición
Cambios de gobierno	Cambios en las autoridades gubernamentales en los niveles nacionales, departamentales y municipales, gracias a los procesos democráticos de elección popular que se gestan cada cuatro años en Colombia
Pandemia como obstáculo	Papel desempeñado por la pandemia en generar barreras o limitaciones para una adecuada implementación de la política pública
Capacidades territoriales	Recursos, habilidades y fortalezas que tienen los municipios y los departamentos para formular o ejecutar acciones en sus territorios por parte de sus gobiernos
Búsqueda de integralidad en salud	Interés y necesidad por alcanzar acciones que integren los diferentes componentes de la atención en salud, a nivel individual y poblacional
Ausencia de lineamientos	Falta de indicaciones o de instrucciones que orienten la implementación de la política en los territorios
Avances en la implementación	Descripción de los logros parciales que se han hecho sobre la ejecución total o parcial de la política o de alguno de sus componentes establecidos en sus modelos operacionales, tanto anteriores como actuales

## RESULTADOS

### Cambios de gobierno

Los cambios de gobierno, tanto nacionales como territoriales, influyen en la implementación de políticas públicas como la PAIS. Un nuevo gobierno puede mantener pero también suspender o incluso cambiar decisiones políticas que venían desarrollándose previamente. Por ejemplo, desde la perspectiva del MSPS en relación con la PAIS: "en el 2018 con el cambio de gobierno pues se evalúa un poquito qué tanto se había avanzado en eso y digamos que sí había avanzado pero muy poco, unos territorios un poco más que otros" (Entrevista MSPS-b, 2021). Para el Ministerio, el balance no era positivo y se requerían cambios en la implementación.

Uno de los argumentos para cambiar el Modelo Integral de Atención en Salud (MIAS), vigente entre el 2016 y el 2019, por el MAITE, era que: "el modelo si es muy interesante, muy completo, pero aterrizarlo no es tan fácil y de pronto es un modelo adecuado para países mucho más robustos y más maduros en todo el tema de su seguridad en salud" (Entrevista MSPS-a, 2021). Lo anterior concuerda con el PND 2018-2022, que reconoce que no todas las ET tenían las capacidades técnicas y operativas para ejecutar integralmente las acciones que proponía el MIAS 15, lo que justificó su cambio.

Adicionalmente, "el cambio de gobierno significa mucho, hasta borrar muchas de las acciones, y toda la pedagogía y asistencia que se ha tenido con las entidades se borran cuando llegan nuevos gobiernos" (Entrevista MSPS-a, 2021). Este tipo de procesos, que son usuales dentro del ejercicio político y democrático propio de nuestro país, se traducen en retos importantes para la implementación y la continuidad de las políticas públicas.

A nivel territorial, los cambios de gobierno se presentaron a comienzos del año 2020, y estas circunstancias influyeron también sobre el proceso de implementación de la PAIS. Desde la SSC se comentó que: "se da un proceso de transición de cambio de nuevo gobernador, de gabinete; y por supuesto por la pandemia. Todos estos momentos coyunturales por decirlo así, quedó en standby [SIC], el tema de la continuidad e implementación de la ruta de mantenimiento y promoción de la salud, o del proceso acorde con el marco metodológico como tal" (Entrevista SSC, 2021). De esta manera, el cambio de gobierno en las ET generó una suspensión temporal de la ejecución de esta política, específicamente en cuanto a las rutas integrales de atención en salud (RIAS).

De acuerdo con el MSPS, el cambio del MIAS al MAITE ha requerido el acompañamiento y la comunicación para reposicionar la política en los territorios: "se fue trabajando con los territorios, se hicieron encuentros con los diferentes secretarios de salud, aquí en Bogotá, se fueron desarrollando como este trabajo de incluir a todos los actores llámense asociaciones de usuarios, en EPS [Entidades Promotoras de Salud], IPS [Instituciones Prestadoras de Salud] para qué, para llegar en cada territorio a un diagnóstico a unos problemas claramente ya identificados" (Entrevista MSPS-b, 2021). Con posterioridad este proceso quedó suspendido con los cambios de gobiernos locales, lo que implicaba volver a posicionar la PAIS y el MAITE con los nuevos gobernantes.

### La pandemia como obstáculo en la implementación

De acuerdo con el MSPS, "básicamente pues la pandemia nos deja a todos, nos lleva básicamente a trabajar en función de lo mismo, el resto de actividades como que quedan ahí pendientes, o sea, en ese momento hablar de temas tan complejos de estos en medio de una pandemia pues no funcionaba y los recursos existentes había que, y necesariamente tenían que moverlos en función y resolver todos estos problemas" (Entrevista MSPS-b, 2021). Estas circunstancias pausaron las acciones relacionadas con la ejecución de la PAIS. A diferencia de los cambios de gobierno, la pandemia era una situación no esperada, pero que afectaría su implementación.

### Capacidades territoriales para la implementación

Las ET asumen la implementación de políticas como PAIS con sus propias capacidades y recursos. Por ejemplo, "el territorio le dice a uno: oiga, es que mire mis recursos humanos o sea son recursos limitados y digamos nos disparan desde diferentes partes y el territorio trata de hacer lo mejor de sí, pero pues con todas estas limitaciones se enreda en todo esto" (Entrevista MSPS-b, 2021). En este sentido, y como ejemplo de lo anterior, desde la Secretaría de Salud de Ubaté, se expresó: "En la provincia tenemos, digamos, son 10 municipios que la conformamos de los 10 digamos que solamente Ubaté es el que tiene una infraestructura para poder darle atención a la comunidad, pero digamos los servicios no logran suplir las necesidades la comunidad" (Entrevista Ubaté, 2021). Esto muestra las dificultades en la atención en salud en esta ET, debido a las necesidades que superan la capacidad de respuesta de este territorio para ofrecer servicios adecuados en salud para la población.

En cuanto a otros de los municipios, se comentó: "por la cantidad de población que tiene Sibaté, son casi 40 000 habitantes, y a nivel de, digamos del plan [territorial de salud], contar nada más de pronto con tres auxiliares que son las que hacen la caracterización principal de la población es poquita gente, son muy poquitas para 40 000, una sola jefe pues dentro del plan y también es muy poquito" (Entrevista Sibaté, 2021). En este municipio se resaltó como una debilidad, en cuanto a sus capacidades, el personal de salud insuficiente para algunas de las acciones en salud que se desarrollan en el municipio. Inclusive, la funcionaria mencionó que es fundamental contar con más talento en salud para lograr ejecutar las acciones que propone el MAITE.

### Búsqueda de la integralidad en salud

Un aspecto relevante que se comentó, por parte de la Secretaría de Salud de Facatativá, fue: "... y apoyar más a la población en determinar cuáles son los riesgos que tiene para su salud, pero no solamente física, la que siempre se dice, la emocional, la psicosocial, social, y el poder ayudarle avanzar, entonces nuestro éxito en esta política, en este plan de desarrollo, con la política de PAIS y con el MAITE, es que nos, nos hace que sea integral, y que integre a las otras, digamos, que sectores, o sea, las otras secretarías" (Entrevista Facatativá, 2021). Este planteamiento refleja, por un lado, el esfuerzo por integrar las diversas dimensiones de la salud, física, emocional, psicosocial y social, y por otro, integrar a otros ámbitos o sectores, en cabeza de otras secretarías de este municipio, y esto es fundamental desde la perspectiva de los determinantes sociales de la salud y la acción intersectorial.

Sin embargo, esta misma participante menciona que "falta más esa integralidad entre esos tres subprogramas [salud pública, aseguramiento y prestación de servicio], que eso es lo que hemos querido hacer, y es lo que es débil en este momento, y se tiene que fortalecer en estos dos años y medio que nos faltan" (Entrevista Facatativá, 2021). Es decir, la integralidad se visualiza como un reto, no solo desde la búsqueda de la acción intersectorial, sino también desde adentro del mismo sector, donde puede ser común la falta de articulación. Este es un reto fundamental en términos del objetivo de la PAIS y el MAITE, especialmente porque contienen el término Integral como parte fundamental de la atención en salud a las personas.

En Sibaté también se reconoció que "se trabaja muy individual a nivel de salud, lo de salud es como muy cerrado pero muchas otras secretarías pueden brindar espacios y otro tipo de pronto otra mirada que nos puede ayudar a implementar de pronto la política desde otro ángulo y llegar de otra manera a la población porque, pues lo que yo te decía, tenemos aquí lo que es grupos poblacionales, pero eso no le pertenece de pronto como tal a la secretaría de salud sino a secretaría de desarrollo social" (Entrevista Sibaté, 2021). Aquí la integralidad, que parece ir de la mano con intersectorialidad, refleja un reto para los territorios debido a la forma en que se distribuyen las competencias y las responsabilidades desde el Estado.

### Ausencia de lineamientos para la implementación

El MAITE no cuenta aún con una guía o lineamiento de implementación que oriente su ejecución en los territorios. Este lineamiento fue propuesto en la Resolución 1147 de 2020 16, con la que amplía los plazos para la formulación de los planes de acción e implementación (PAIM) del MAITE; estos planes permiten, de acuerdo con esta norma, la construcción y puesta en marcha de este modelo. Desde el MSPS se comentó lo siguiente: "la resolución dice: vamos a trabajar en una guía, la resolución no define específicamente cuando el Ministerio va a entregar la guía, no da una fecha, pero lo que sí dice es que los territorios tendrán un año a partir de qué se les entregué oficialmente la guía" (Entrevista MSPS-b, 2021).

Desde el departamento de Cundinamarca, se percibe así la ausencia de un lineamiento o guía para la implementación del MAITE y su plan de acción: "el Ministerio durante el año 2019 sacaron como una estructura muy básica para formular un plan de acción para MAITE en el Departamento de Cundinamarca y algunas acciones, pero pues realmente esto no tenía unos lineamientos claros en cada línea de acción del MAITE" (Entrevista SSC, 2021).

Luego, se añadió: "Decidimos esperar [...] efectivamente el Ministerio dijo: vamos a sacar unos lineamientos para la formulación del proyecto del plan de acción de MAITE, entonces hasta que nosotros nos saquemos esos lineamientos pues prácticamente no hacemos nada, o sea, estamos en standby [SIC]" (Entrevista SSC, 2021). Desde la SSC se considera que es mejor esperar para que estos planes se formulen bajo los mismos criterios para todas las ET. De esta manera, la ausencia de este lineamiento se convierte en otro elemento que ha retrasado la implementación de la PAIS en el departamento.

### Articulación con la planeación territorial en salud

Para el MSPS, el MAITE es un instrumento o herramienta para la planeación en salud en las ET. Una de las apreciaciones fue: "si esto [el MAITE] va a ser un tema de planeación para ayudarle al territorio tiene que ser una herramienta clara, concisa, que le dé mapa visual de cómo se va a mover o moverse" (Entrevista MSPS-b, 2021). Y luego, se complementó con los siguiente: "O sea porque éstas son muchas herramientas, como muchos lineamientos para planeación del territorio, pero cómo que todas sí, pero a la vez no hay articulación no se hablan al final es lo mismo, y realmente las visitas que hicimos al territorio y lo digo con digamos desde la experiencia con los territorios que yo estuve la preocupación de los territorios es Dios mío, más tareas, más cuadros, más matrices" (Entrevista MSPS-b, 2021). Uno de los desafíos más importantes es lograr una adecuada articulación y coordinación con los mecanismos de planeación ya existentes, que no implique una mayor carga laboral o, incluso, la duplicidad de acciones.

Otra de las funcionarías del MSPS planteó: "¿cómo es que vamos a armonizar el MIAS, el MAITE con la planeación territorial?, ¿cómo alinear al interior de la planificación territorial, alinear los diferentes instrumentos que ellos tienen como es el ASIS [Análisis de Situación de Salud], ¿la caracterización y priorización? ¿cómo la vamos a organizar para que sirva para la planeación?" (Entrevista MSPS-a, 2021). Este punto es fundamental porque sus interrogantes revelan que la planeación en salud en las ET cuenta con diferentes herramientas e instrumentos y el reto es lograr que operen armónicamente. Por consiguiente, un instrumento, como desde el MSPS se considera el MAITE, implica que sus capacidades deben ajustarse a esta.

Las ET ejecutan otros instrumentos de política para la planeación en salud, como el Plan Decenal de Salud Pública, el PTS y otros de diferentes sectores que se encuentran planteados en sus planes de desarrollo. Por ejemplo, se destaca que: "desde el Plan Decenal en Salud Pública, además de toda la construcción de metas y eso, se está haciendo un proceso de armonización con todos los instrumentos de planeación y políticas para empezar a mirar dónde es el punto de encuentro de ellos y cómo es que los vamos a empezar armonizar para que todos caminen de la mano y cómo todos van a quedar dentro del plan" (Entrevista MSPS-a, 2021). Esta armonización por tanto incumbe a un instrumento como el MAITE.

También se afirmó que "tenemos que apostarle principalmente a eso, a poder definir realmente cuál va a ser la manera en qué territorio va a planear sus acciones en salud, y en ese momento ahí si la herramienta tiene mucho sentido no, porque eso se está yendo como de la mano, en ese momento si logramos sacar esta herramienta sería para aplicarla ya en los territorios y que los territorios arranquen" (Entrevista MSPS-b, 2021). Este planteamiento expresa la necesidad de contar con un lineamiento claro y operativo para la ET, que se integre adecuadamente a la planeación en salud a nivel local, y esto parece que aún no se ha definido todavía.

Desde la visión departamental se tiene en cuenta esta armonización de instrumentos de planeación territorial, que finalmente se convierten en decisiones de política pública para sus territorios: "Las RIAS [Rutas Integrales de Atención en Salud] van encaminadas a MAITE, MAITE va encaminado al Plan decenal de Salud Pública, así de sencillo, y que unificarán herramientas instrumentos para presentar los debidos planes el plan de MAITE y los informes de ese marco entonces, digamos que estamos en este proceso de transición y entendimiento" (Entrevista SSC, 2021).

### Avances en la implementación

Se identificaron acciones que han mostrado algunos avances desde el contexto territorial en Cundinamarca. Dichas acciones hacían parte del anterior MIAS y siguen sosteniéndose en la actualidad a pesar de que este modelo fue derogado y el MAITE no tiene un lineamiento general para su implementación. Estas son la caracterización poblacional, las rutas integrales de atención en salud (RIAS) y las redes integradas de servicios de salud (RISS) 17.

Frente a la primera, desde el MSPS se comentó lo siguiente: "como caracterización podríamos decir que es el ASIS, porque el ASIS es un perfil y un análisis epidemiológico de tipo ya poblacional que incluye todas las situaciones salud que hay en los territorios entonces ahí además de haber en indicadores en salud y mortalidad, también tenemos algunos comportamientos, condiciones y las características sociodemográficas poblacionales y algunos determinantes sociales de la salud" (Entrevista MSPS-b, 2021). Esto es importante porque se considera que el ASIS, tarea que deben hacerse tanto a nivel municipal como departamental, se constituye en un componente esencial de la PAIS.

Con relación a las RIAS, divididas en aquellas para la promoción y mantenimiento de la salud, para poblaciones en riesgo y para eventos específicos priorizados en estas poblaciones 11, cuentan con una reglamentación específica que se ha venido desarrollando desde el 2016, no solo con la Resolución 429 sino también con la Resolución 3202 que establece el Manual metodológico para su elaboración e implementación, siendo obligatorias para todas las ET 17,18. Además, en el 2018 se promulgaron, a través la Resolución 3280, los lineamientos operativos y las directrices de estas rutas, especialmente para la promoción de la salud y para la atención mater-no-perinatal, también de obligatorio cumplimiento para los actores del SGSSS, incluidas las ET 19.

Al respecto, desde la SSC se explicó: "el departamento de Cundinamarca adoptó el modelo metodológico de la 3202, para la implementación de las RIAS de mantenimiento y promoción de la salud y de la ruta materno perinatal en el marco del MIAS, no en ese momento, en el marco del MIAS que es el modelo que propuso PAIS, digamos en el marco del MIAS como modelo de implementación" (Entrevista SSC, 2021). Estos lineamientos a nivel metodológico pueden contribuir a explicar la razón por la que este componente de la PAIS ha logrado avanzar en el departamento. Adicionalmente, se comentó: "Se montó un plan de acción para la implementación de la ruta de mantenimiento y promoción de la salud y, empezaron a hacer esas actividades cada dirección hacía, acorde con su competencia" (Entrevista SSC, 2021).

En Sibaté se destacó el avance en la implementación de estas rutas: "hemos logrado sacar todas las RIAS enfocadas a salud mental, todo lo de violencias, todo lo de, bueno, todo salud mental, SPA, que otra cosa, ideaciones suicidas y ahorita estamos es actualizando la ruta de materno perinatal a nivel de ente territorial" (Entrevista Sibaté, 2021). Aquí mencionan diferentes RIAS que han logrado formular en diferentes ámbitos conforme a como es exigido en la normatividad, especialmente la Resolución 3202 del 2016 18.

Un tercer componente de la PAIS que se ha identificado en el contexto departamental y que cuenta también con algunos avances lo constituyen las redes integradas de servicios de salud (RISS), contenidas en el MIAS 17. En Cundinamarca, gracias la Ordenanza 07 del 2020, se dispuso la reorganización y modernización de la red pública de prestadores de servicios de salud del departamento 20. De esta forma, se organizaron 14 regiones de salud que integran los 116 municipios y cuentan con un año a partir de la expedición de la norma como período de transición.

En la Secretaría de Salud de Ubaté, en torno a esta decisión política se manifestó: "en redes digamos que nuestro hospital sería el mayor beneficiado una vez inicie la implementación cómo estás de esas mismas redes, digamos, entonces que eso ayudaría también pues en la implementación de la PAIS" (Entrevista Ubaté, 2021). Se valora positivamente la implementación de las RISS en el departamento, teniendo en cuenta que este municipio cuenta con un hospital de segundo nivel que debe cubrir no solo la población de su territorio sino de otros nueve que hacen parte de la misma provincia: "Ubaté es el que tiene una infraestructura para poder darle atención a la comunidad pero digamos los servicios no logran suplir las necesidades de la comunidad entonces los demás municipios tenemos puestos de salud pues muchas de las personas llegan a Ubaté" (Entrevista Ubaté, 2021). Para la SSC, es una ventaja contar con redes organizadas y modernizadas "para que no pongan a los pacientes a hacer el paseo de la muerte o digamos que cada red tenga los servicios acordes con su demanda y no se saturen" (Entrevista SSC, 2021).

## DISCUSIÓN

El estudio tuvo como objetivo principal caracterizar la situación actual de implementación territorial de la PAIS, por medio de un estudio del caso en el departamento de Cundinamarca, Colombia, para el año 2021. Los resultados, basados en un diseño cualitativo cuyo instrumento principal de recolección de información fue la entrevista individual semiestructurada, muestran que este proceso ha tenido algunos elementos característicos reflejados en los temas identificados y generados en el análisis. En el ámbito nacional, la llegada del nuevo gobierno en el año 2018 trajo consigo el cambio en el modelo operacional de la PAIS. Fue derogado el MIAS y se adoptó el MAITE, instrumento que pretende adaptarse mejor a las condiciones y las realidades locales de muchos municipios en el país y lograr así los objetivos de la política.

Además, el cambio de gobierno a nivel territorial, en los departamentos, municipios y distritos en el 2020, contribuyó también a que se afectara la implementación de la PAIS; con los nuevos gobernantes y sus equipos de trabajo se requería el reposicionamiento del tema en las agendas políticas locales para que se incorporara a sus PTS. Y, cuando aún se estaba realizando el acompañamiento desde el MSPS a los departamentos y municipios sobre los cambios en la PAIS y su modelo operacional, llegó la pandemia y obligó a la suspensión de la mayoría de las acciones en los territorios. Los planes de desarrollo y los PTS tuvieron que ajustarse a la nueva realidad traída por las condiciones epidemiológicas propias del covid-19.

El estudio realizado por Fachel *et al.*^21^ reconoce que las brechas entre la formulación y la implementación de una política pública son inevitables. Los obstáculos a nivel institucional y político, así como los recursos limitados, contribuyen a la existencia de estas brechas entre lo que se espera de una decisión política y lo que finalmente se puede lograr. Estos planteamientos coinciden con la realidad política alrededor de la PAIS y su implementación territorial, en nuestro caso de Cundinamarca.

Por otra parte, la investigación realizada por Bezerra *et* al. 22 sobre la implementación de la Política Nacional de Atención Integral en Salud en Adolescentes privados de la Libertad, destaca que esta iniciativa fortalece la realización del derecho a la salud de estos grupos poblacionales, pero falta promoverse y mejorarse su implementación en los estados y municipios brasileños. Al igual que el estudio citado anteriormente, la implementación de estas políticas se convierte en un reto cuando deben ejecutarse a nivel territorial.

El trabajo de Vollrath *et al.*^23^, alrededor de los desafíos de la implementación del sistema de protección infantil de Chile, resalta que la política enfrenta un proceso complejo de cambio donde la política enfrenta barreras administrativas y de personal, así como desafíos en el trabajo interdisciplinario y las redes intersectoriales, aspectos fundamentales en una política de carácter participativo e inclusivo. Por lo tanto, la investigación refleja que la implementación de políticas se encuentra con obstáculos y limitaciones en las capacidades institucionales y realidades locales, como sucede en el caso colombiano con la PAIS.

En Brasil, Neves *et al.*^24^ afirman que el sistema de salud de este país desde la década de 1990 ha buscado la integralidad en los servicios, con el fin de garantizar el acceso a estos por parte de toda la población, incluyendo la articulación entre acciones individuales y colectivas, preventivas, promocionales y curativas. Por otra parte, Santos *et al.*^25^ consideran que la atención integral en salud presenta desafíos importantes, debido a la escasez de oferta de profesionales especializados, la fragmentación de la red de servicios y la desarticulación a nivel comunicativo entre los niveles del sistema de salud brasileño. Algunos análisis y estudios en este país, por consiguiente, muestran la importancia de la integralidad en salud, pero también los retos que presenta en términos de su ejecución.

Dos últimos puntos que considerar son los siguientes: el primero es la planeación territorial en salud. En Colombia se cuenta con la Resolución 1536 del 2015 26, que establece sus disposiciones más relevantes. En su contenido, la planeación en salud en los departamentos y municipios toma como referentes de política el Plan Decenal de Salud Pública y los Análisis de Situación de Salud para la elaboración de los PTS. Sin embargo, no se incluye la PAIS ni su modelo operacional, lo que representa un vacío que es preciso llenar para lograr una adecuada articulación entre las políticas y que la planeación territorial pueda ser más eficiente y efectiva. El segundo punto es el lineamiento ausente para la implementación del modelo operacional de la PAIS, es decir, el MAITE. La Resolución 2626 del 2019 12, que creó este modelo, estableció también la formulación y puesta en marcha de un plan de acción e implementación por parte de las ET con el acompañamiento del MSPS; sin embargo, a la fecha del estudio, ni este plan ni sus lineamientos habían sido formulados porque el territorio se encontraba a la espera de ellos desde la autoridad nacional en salud. La ausencia de este lineamiento dificulta también la continuidad esperada en la ejecución de la PAIS a nivel territorial.

Este estudio tuvo como limitación que, debido a las condiciones restrictivas de la pandemia, pocos municipios del departamento tuvieron disponibilidad de tiempo para participar. Sin embargo, el estudio logró hacer una importante aproximación, contribuyendo a la generación de evidencia sobre los procesos de implementación de políticas públicas en salud, siendo esta su principal fortaleza.

Como conclusión, la PAIS y su modelo operacional tienen importantes desafíos para su implementación desde los territorios, donde deben enfrentar situaciones como los cambios de gobierno, sus limitadas y diferentes capacidades para actuar conforme a las decisiones políticas y, al mismo tiempo, resolver las necesidades en salud que tienen. Es importante continuar con el desarrollo de otras investigaciones que analicen la implementación de la PAIS y el MAITE en otras ET, tanto departamentales como municipales, a nivel de la política general y también tomando como énfasis algunas de sus líneas de acción o componentes, como las RIAS o las RISS, la gobernanza o la intersectorialidad ♠
